# Prevention of Infantile Paralysis

**Published:** 1916-08

**Authors:** 


### Prevention of Infantile Paralysis.

To control the present epidemic of infantile paralysis, according to a statement issued by the United States Public Health Service today, the chain of infection between persons harboring germs of the disease and the well members of the community should be broken. Infantile paralysis is probably caused by a very minute organism found in the nasal, mouth and bowel discharges of those who have the disease or who are carriers of the germ without themselves suffering from the ailment. All of the steps in the spread of the infection are not known, but if this germ can be prevented from passing from the infected to the well person, the disease will cease.

Infantile paralysis is not a disease of recent origin. Sporadic or scattered cases have occurred throughout the country for many years, but it is only during the last decade that the infection has assumed epidemic proportions in the United States. The present epidemic in New York City, on account of its magnitude and virulence, has awakened the residents of many communities to the danger of the importation of the disease into their own midst. This danger is real, but if due precautions are exercised it is believed that the epidemic will subside.

The actual control of the present epidemic must be left to the city, State and Federal health authorities. These organizations will properly quarantine and care for affected persons, prescribe sanitary measures and limit as may be necessary the travel of individuals in order to protect neighboring districts from the infection. Individuals and communities, however, can do much toward their own protection.

Poliomyelitis is probably spread directly or indirectly through the medium of infective secretions. Account must, therefore, be taken by communities of every means by which such secretions are disseminated. Promiscuous expectoration should be controlled. The common drinking cup affords a method for the interchange of material of this nature and should, therefore, be abolished.

Rigid cleanliness of glasses and utensils at soda fountains, in saloons and other public places should be enforced. Flies, roaches and other vermin, by coming in contact with infective secretions, may possibly convey them to our food and thus directly bring about the development of disease. Therefore eliminate insects. Street and house dust bear a definite relation to the spread of many infections, and it is not unreasonable to presume that they may be a factor in the dissemination of infantile paralysis. Maintain strict cleanliness of streets, yards and alleys in order to prevent the breeding of insects and other vermin. See that all garbage and waste are properly cared for and collected at regular and frequent intervals. Guard all food supplies, especially milk and other perishable products. Digestive troubles of children arising from the ingestion of food of questionable quality may lower resistance. Assemblies of children in infected localities are to be discouraged, if not actually forbidden. While the above measures are in a sense general, and applicable to many epidemic diseases, their importance should not be overlooked.

Individual preventive measures may be thus summarized:

Summon a physician at once and immediately notify the health officer of the presence of the disease. If the disease is present in the community, medical aid should be sought whenever a child is sick, no matter how light the illness; many cases of infantile paralysis begin with a slight indisposition. Should the illness prove to be infantile paralysis isolate the patient, place a competent person in charge, and reduce all communication with the sick room to a minimum. Hospital care is preferable, not only for the child but in order to better safeguard against the spread of the disease. The sick room should be well ventilated and screened. Nasal and mouth secretions should be received in cloths, placed in a paper bag, and burned. The clothing of the child, the bed linen, and the excretions should be disinfected in the same manner as for typhoid fever, that is, by boiling, the long continued application of 5 per cent carbolic, or other well recognized disinfectant. The same is true for dishes and drinking vessels. Nurses should exercise the same precautions as regards cleanliness of hands in caring for infantile paralysis patients as for those afflicted with other infectious diseases.

A child may convey the disease to others even after a lapse of several weeks. For this reason quarantine should be maintained for a considerable period, usually from six to eight weeks, and the above precautions should be adhered to during this time. Disinfection of the room following recovery is advisable.

